# Clinical, functional and radiographic outcomes of primary total hip arthroplasty between direct anterior approach and posterior approach: a systematic review and meta-analysis

**DOI:** 10.1186/s12891-020-03318-x

**Published:** 2020-06-02

**Authors:** Linbo Peng, Yi Zeng, Yuangang Wu, Junfeng Zeng, Yuan Liu, Bin Shen

**Affiliations:** 1 Department of Orthopaedic Surgery, West China Hospital, West China Medical School, Sichuan University, Chengdu, Sichuan Province 610041 People’s Republic of China; 2 Department of Orthopedics, West China Hospital, Sichuan University, 37# Guoxue Road, Chengdu, 610041 People’s Republic of China

**Keywords:** Total hip arthroplasty, Surgical approach, Direct anterior approach, Posterior approach, Early functional recovery

## Abstract

**Background:**

The purpose of this systematic review and meta-analysis was to compare the direct anterior approach and posterior approach for primary total hip arthroplasty in terms of the clinical, functional and radiographic outcomes.

**Methods:**

We searched the PubMed and EMBASE databases and Cochrane Library from their inception to November 1, 2019. We searched for previously published articles and meta-analyses of randomized controlled trials.

**Results:**

A total of 7 randomized controlled trials with 600 participants met the inclusion criteria. Among these patients, 301 and 299 were included in the DAA and PA groups, respectively. The DAA was associated with a longer surgery by a mean duration of 13.74 min (95% CI 6.88 to 20.61, *p* < 0.0001, I^2^ = 93%). The postoperative early functional outcomes were significantly better in the DAA group than in the PA group, such as the Visual Analogue Scale (VAS) score at 1 day postoperatively (MD = -0.65, 95% CI − 0.91 to − 0.38, *p* < 0.00001, I^2^ = 0%), VAS score at 2 days postoperatively (MD = -0.67, 95% CI − 1.34 to − 0.01, *p* = 0.05, I^2^ = 88%) and Harris Hip Score (HHS) at 6 weeks postoperatively (MD = 6.05, 95% CI 1.14 to 10.95, *p* = 0.02, I^2^ = 52%). There was no significant difference between the DAA and PA groups in the length of the incision, hospital length of stay (LOS), blood loss, transfusion rates or complication rates. We found no significant difference between the two groups regarding late functional outcomes, such as the VAS score at 12 months postoperatively or the HHS scores at 3, 6, and 12 months postoperatively. A significant difference in the radiographic outcomes was not detected.

**Conclusions:**

The DAA requires a longer surgery time than does the PA in primary total hip arthroplasty. The DAA yields better early functional recovery than does the PA. There was no significant difference between the two groups in terms of other clinical, complication-related, late functional or radiographic outcomes. The evidence on the superiority of the DAA is insufficient and needs to be studied further.

## Background

With the aging of society, the morbidity of knee and hip osteoarthritis is increasing rapidly, causing a large social and economic burden [1, 2]. Total hip arthroplasty (THA) is the gold standard treatment for end-stage OA [3]. THA surgery has greatly improved the functional status of patients over the last half-century [4].

Many surgical approaches are used in THA surgery, but there is little evidence indicating which approach has the most advantages [5, 6]. On the one hand, the direct anterior approach (DAA) is considered a true minimally invasive approach because it leads to a small amount of muscle damage, as the operation is performed through a small incision and a muscle interval in the hip joint [7, 8]. On the other hand, the conventional posterior approach (PA) is the most frequently used surgical approach for THA [9].

Some studies show that compared with the PA, the DAA leads to less blood loss, low transfusion rates, shorter surgery times, a shorter length of hospital stay (LOS), low postoperative complication rates and better functional recovery [10–17]. Other studies have shown that DAA is associated with higher postoperative complication rates than is PA [18, 19], especially regarding neuropraxia in the lateral cutaneous nerve of the thigh [18, 20]. Several meta-analyses have been published, but their results are not enough to be convincing. Retrospective studies and non-randomized controlled trials are included in most of the meta-analyses, which leads to indirect evidence [21–23]. Nonstandard approaches such as piriformis preserving approaches and those involving computer-aided technology were inappropriately regarded as standard approaches in some studies [21–25], which should be strictly avoided for accurate results. Therefore, we performed a meta-analysis with strict inclusion criteria and includes the most recently published RCTs to compare the direct anterior approach and posterior approach for primary total hip arthroplasty in terms of the clinical, functional and radiographic outcomes.

## Methods

### Search strategies

We performed this study in accordance with the Cochrane Handbook for Systematic Reviews of Interventions [26] and Preferred Reporting Items for Systematic Reviews and Meta-Analyses (PRISMA) guidelines [27]. We searched the PubMed and EMBASE databases and Cochrane Library from their inception to November 1, 2019. We searched for previously published articles and meta-analyses of randomized controlled trials. We used the keywords “Arthroplasty, Replacement, Hip” and “approach” to identify published RCTs, and we did not use any language restrictions.

The following electronic search strategy was used for PubMed: ((((((((((randomized controlled trial [pt]) OR controlled clinical trial [pt]) OR randomized [tiab]) OR placebo [tiab]) OR clinical trials as topic [mesh: noexp]) OR randomly [tiab]) OR trial [ti])) NOT ((animals [mh] NOT humans [mh])))) AND ((approach [Title/Abstract]) AND ((((((((((((((((((((“Arthroplasty, Replacement, Hip”[Mesh]) OR Arthroplasties, Replacement, Hip [Title/Abstract]) OR Arthroplasty, Hip Replacement [Title/Abstract]) OR Hip Prosthesis Implantation [Title/Abstract]) OR Hip Prosthesis Implantations [Title/Abstract]) OR Implantation, Hip Prosthesis [Title/Abstract]) OR Implantations, Hip Prosthesis [Title/Abstract]) OR Prosthesis Implantation, Hip [Title/Abstract]) OR Prosthesis Implantations, Hip [Title/Abstract]) OR Hip Replacement Arthroplasty [Title/Abstract]) OR Replacement Arthroplasties, Hip [Title/Abstract]) OR Replacement Arthroplasty, Hip [Title/Abstract]) OR Arthroplasties, Hip Replacement [Title/Abstract]) OR Hip Replacement Arthroplasties [Title/Abstract]) OR Hip Replacement, Total [Title/Abstract]) OR Replacement, Total Hip [Title/Abstract]) OR Hip Replacements, Total [Title/Abstract]) OR Replacements, Total Hip [Title/Abstract]) OR Total Hip Replacements [Title/Abstract]) OR Total Hip Replacement [Title/Abstract])).

### Eligibility criteria

1. Participants: patients undergoing primary THA;
2. Interventions: the intervention group underwent THA surgery with the DAA;
3. Comparisons: the control group underwent THA surgery with the PA;
4. Outcomes: clinical outcomes such as the length of the incision, surgery time, length of hospital stay, blood loss, and transfusion rates; complications such as dislocation, fracture, LCNT neuropraxia, DVT and overall complications; radiographic outcomes such as acetabular inclination and acetabular anteversion; functional outcomes such as the VAS score at 1 day, 2 days, and 12 months postoperatively and the Harris hip score at 6 weeks, 3 months, 6 months, and 12 months postoperatively.
5. Study design: randomized controlled trials.

### Study selection

We imported all the studies identified in the search into Endnote X7 software (Thompson Reuters, CA, USA). Two reviewers (LBP and JFZ) scanned the titles and abstracts independently, and we resolved any disagreements by discussion with senior reviewers. RCTs comparing the DAA and PA in THA surgery were eligible for inclusion. Duplicates were removed, and we also excluded commentaries, letters, case studies and reviews. Nonstandard approaches such as piriformis-preserving approaches, those involving computer-aided technology, or other surgical approaches were also excluded. Then, we read the full texts to exclude other ineligible studies.

### Data extraction

Two authors extracted the following information and then reviewed the information together to guarantee the data were accurate: the name of the first author, publication year, study design, number of surgeons, number of cases in each group, follow-up duration, sex distribution, average age, BMI, learning cases, length of the incision, surgery duration, length of hospital stay, blood loss and transfusion rates, dislocation, fracture, LCNT neuropraxia, DVT and overall complication, acetabular inclination and acetabular anteversion, the VAS score at 1 day, 2 days, and 12 months postoperatively and the Harris hip score at 6 weeks, 3 months, 6 months, and 12 months postoperatively.

### Risk of bias in individual studies

Two authors assessed the risk of bias for each article by the Cochrane Bias risk assessment tool. Disagreements were resolved by discussion with a senior researcher. We determined whether each study had a low, high or unclear risk of bias in each domain.

### Outcome measures and statistical analysis

We conducted this study using Review Manager software 5.3. All the data were extracted into Excel first and then divided into categorical variables and continuous variables. Categorical variables (transfusion rates, complications (such as dislocation, fracture, LCNT neuropraxia, DVT and overall complication)) were expressed as odds ratios (ORs) with 95% confidence intervals CIs. Continuous variables (length of incision, surgery time, length of stay, blood loss, acetabular inclination, acetabular anteversion, the VAS score at 1 day, 2 days, and 12 months postoperatively and the Harris hip score at 6 weeks, 3 months, 6 months, and 12 months postoperatively) were expressed as the mean differences (MD) with 95% CIs. We used a fixed effects model when there was no statistical heterogeneity among the studies (*p* > 0.1, I^2^ < 50%) and a random effects model when heterogeneity existed (*p* < 0.1, I^2^ > 50%). Otherwise, a **descriptive** analysis was used. The results of the meta-analysis were shown in forest plots; we considered *p* < 0.05 to indicate a statistically significant difference.

## Results

### Study selection

**Fig. 1:**
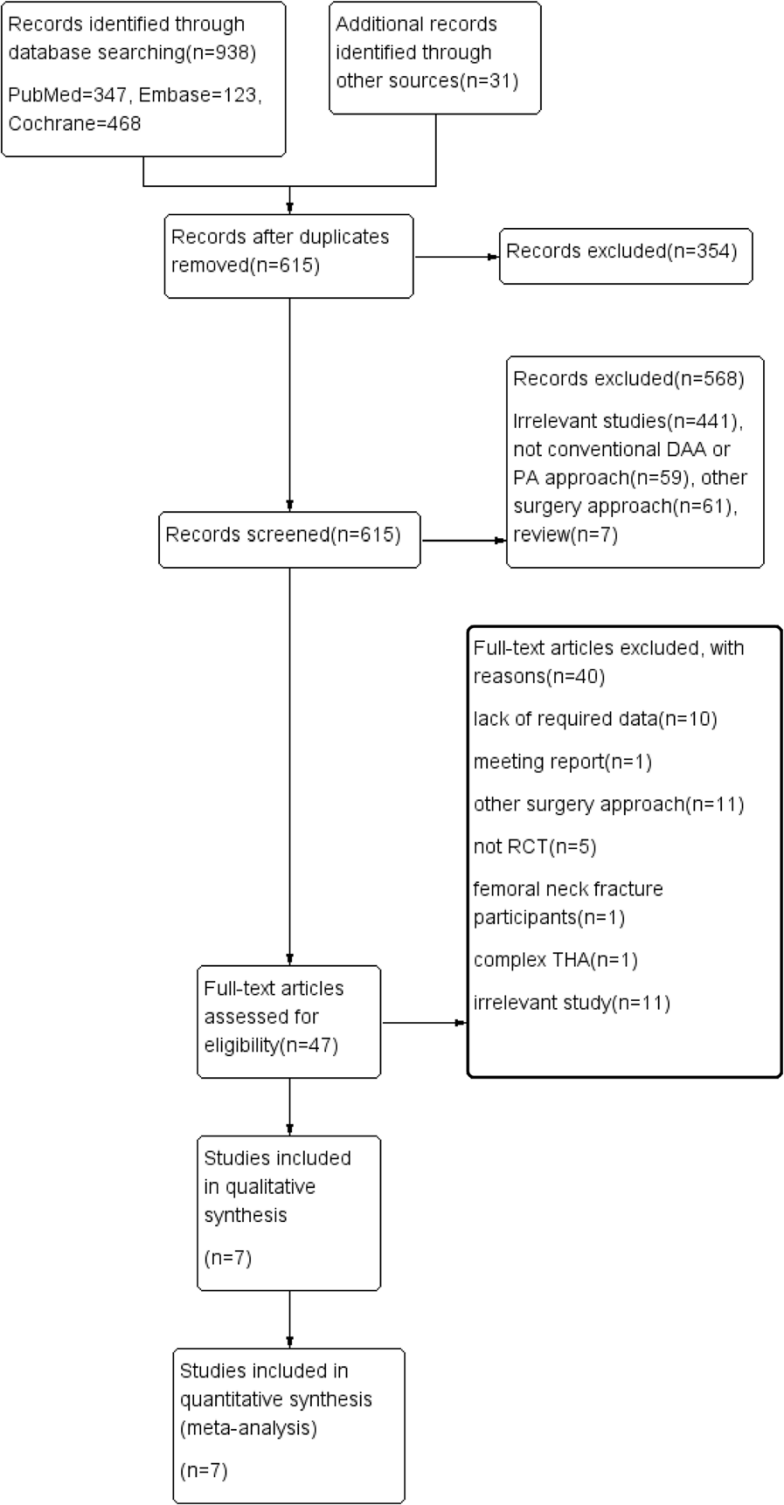
PRISMA study flow diagram

We initially identified 969 studies and included 7 randomized controlled trials with 600 participants in the meta-analysis after screening for eligibility [18–20, 28–31]. The PRISMA study flow diagram was shown in Fig. 1.

### Study characteristics

A total of 7 randomized controlled trials with 600 participants were included. One study showed statistically significant differences between genders [28], and one study did not provide BMI data [19]. None of the studies included learning cases. The demographic characteristics of the patients were shown in Table 1.

**Table 1:** Demographic characteristics of patients

Study	Year	Study design	Surgeon Number	follow-up time	Cases DAA:PA	ages DAA:PA	male/female DAA:PA	BMI DAA:PA	learning cases
Barrett	2013	RCT	1	12 months	43:44	61.4 ± 9.2: 63.2 ± 7.7	29/14: 19/25	30.7 ± 5.4:29.1 ± 5.0	NO
Cheng	2016	RCT	2	12 weeks	35:37	59: 62.5	15/20: 18/20	27.7 28.3	NO
Christensen	2015	RCT	1	6 weeks	28:23	64.3 ± 9.1:65.2 ± 9.1	13/15: 11/12	31.1 ± 5.1:30.4 ± 3.6	NO
Luo	2016	RCT	1	16 months	52:52	61.5 ± 7.2:63.7 ± 6.8	17/35: 22/30	22.7 ± 4.4:24.2 ± 3.7	NO
Rykov	2017	RCT	3	6 weeks	23:23	62.8 ± 6.1:60.2 ± 8.1	8/15: 11/12	29.0 ± 5.6:29.3 ± 4.8	NO
Zhang	2006	RCT	Not clear	30 months	60:60	61: 62.5	25/35: 28/32	not stated	NO
Zhao	2017	RCT	Not clear	6 months	60:60	64.88 ± 12.13:62.18 ± 14.72	24/36: 26/34	24.3 ± 53.1:25.58 ± 2.83	NO

### Risk of bias

**Fig. 2:**
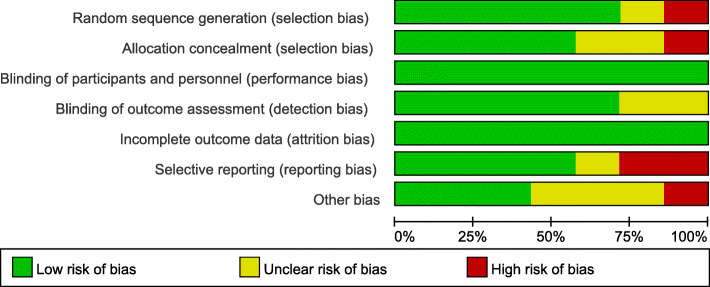
Risk of bias graph: a summary of the authors’ decisions about each risk of bias item presented as percentages across all included studies

**Fig. 3:**
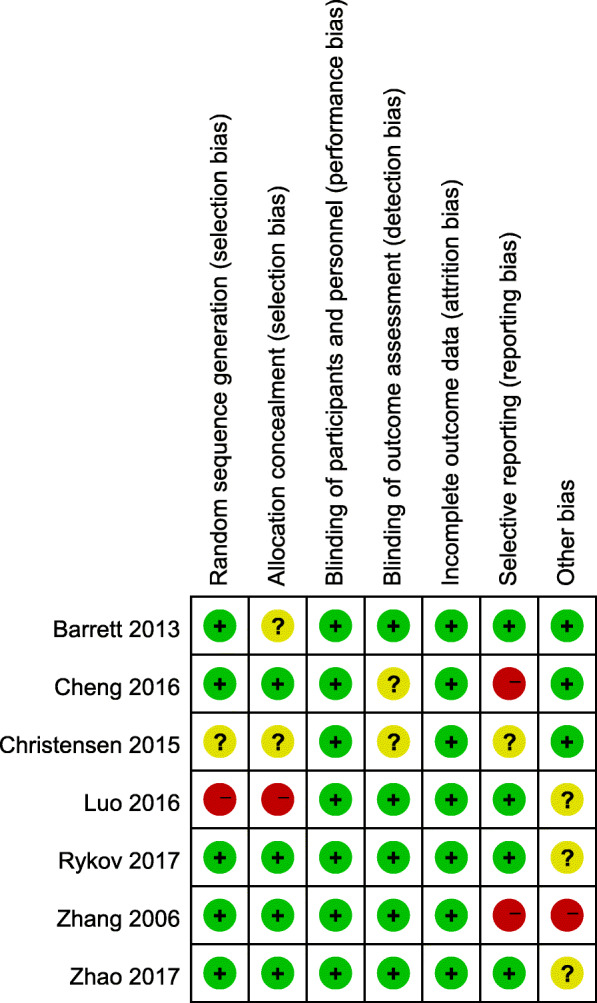
Risk of bias summary: a summary of the authors’ decisions about each risk of bias item for each included study

All the studies included in the meta-analysis were randomized controlled trials of high quality. It is difficult to blind the doctors performing surgeries to the patient groups, but we think that the absence of blinding did not contribute to detection bias, at least in some outcome parameters. The risk of bias graph for each study and the risk of bias summary were shown in Figs. 2 and 3.

### Clinical outcomes

#### Length of the incision

**Fig. 4:**
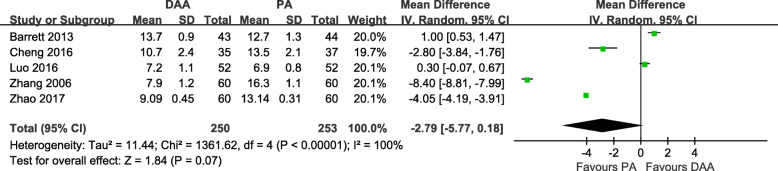
Length of incision (cm) forest plot analysis comparing the DAA vs PA in primary THA

Five studies [18–20, 28, 31] with a total of 503 patients were included in the comparison of the length of the incision between the DAA and PA in primary THA. We failed to find a significant difference between the DAA group and PA group, and there was statistically significant heterogeneity among the studies (MD = -2.79 cm, 95% CI − 5.77 to 0.18, *p* = 0.07, I^2^ = 100%, Fig. 4).

#### Surgery duration

**Fig. 5:**
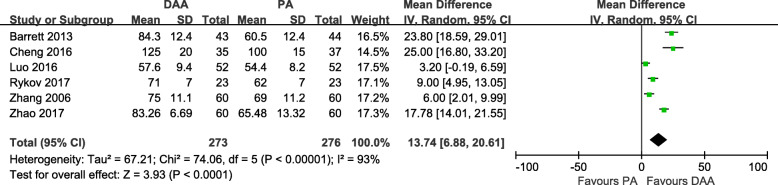
Surgery duration (min) forest plot analysis comparing the DAA vs PA in primary THA

Six studies [18–20, 28, 30, 31] with a total of 549 patients were included in the comparison of the surgery duration between the DAA and PA in primary THA. The DAA required a significantly longer surgery duration (13.74 min, 6.88 to 20.61, *p* < 0.0001, Fig. 5), but there was statistically significant heterogeneity among the studies (I^2^ = 93%).

#### Blood loss

**Fig. 6:**
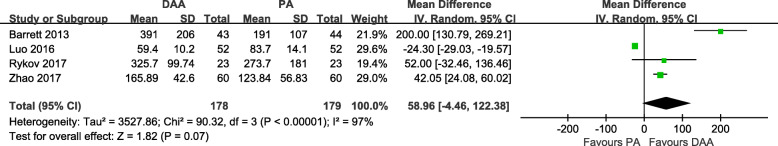
Blood loss (ml) forest plot analysis comparing the DAA vs PA in primary THA

Four studies [20, 28, 30, 31] with a total of 357 patients were included in the comparison of perioperative blood loss between the DAA and PA in primary THA. We failed to find a significant difference between the DAA group and PA group, and there was statistically significant heterogeneity among the studies (MD = 58.96 ml, 95% CI − 4.46 to 122.38, *p* = 0.07, I^2^ = 97%, Fig. 6).

#### Transfusion rates

**Fig. 7:**
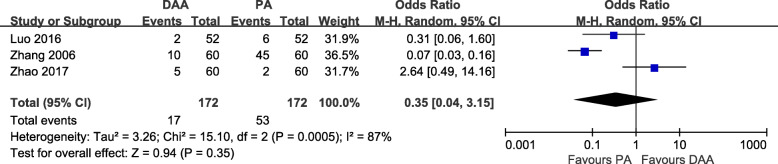
Transfusion rate forest plot analysis comparing the DAA vs PA in primary THA

Three studies [19, 20, 31] with a total of 344 patients were included in the comparison of the transfusion rates between the DAA and PA in primary THA. We failed to find a significant difference between the DAA group and PA group, and there was statistically significant heterogeneity among the studies (OR = 0.35, 95% CI 0.04 to 3.15, *p* = 0.35, I^2^ = 87%, Fig. 7).

#### Length of hospital stay (LOS)

**Fig. 8:**
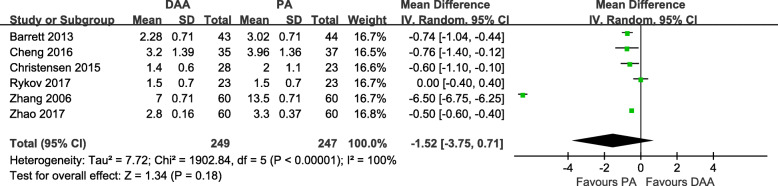
Length of hospital stay forest plot analysis comparing the DAA vs PA in primary THA

Six studies [18, 19, 28–31] with a total of 496 patients were included in the comparison of the LOS between the DAA and PA in primary THA. There was no significant difference between the DAA group and PA group in terms of the LOS (MD = -1.52 day, 95% CI − 3.75 to 0.71, *p* = 0.18, Fig. 8). There was statistically significant heterogeneity among the studies (I^2^ = 100%).

#### Complications

**Fig. 9:**
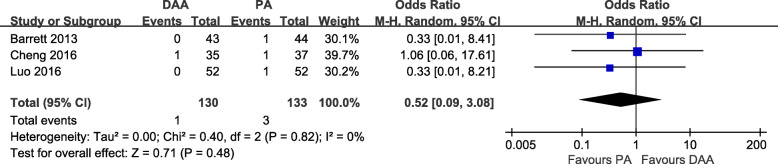
Postoperative dislocation rate forest plot analysis comparing the DAA vs PA in primary THA

**Fig. 10:**
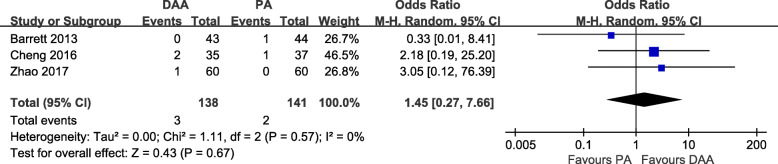
Postoperative fracture rate forest plot analysis comparing the DAA vs PA in primary THA

**Fig. 11:**
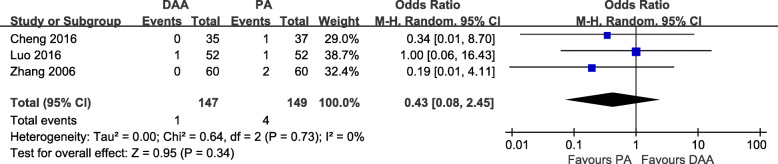
Postoperative DVT rate forest plot analysis comparing the DAA vs PA in primary THA

**Fig. 12:**
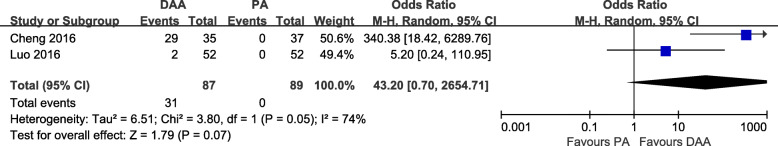
Postoperative LCNT neuropraxia rate forest plot analysis comparing the DAA vs PA in primary THA

**Fig. 13:**
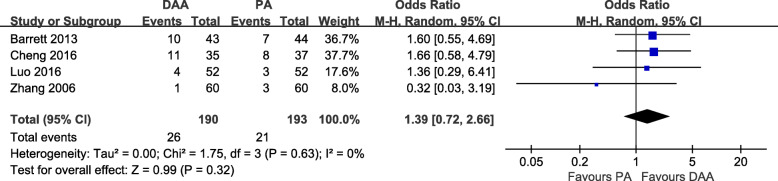
Postoperative overall complication rate forest plot analysis comparing the DAA vs PA in primary THA

Five studies [18–20, 28, 31] were included in the comparison of the complications between the DAA and PA in primary THA. Three studies [18, 20, 28] reported the occurrence of postoperative dislocation. There was no significant difference between the two groups in terms of the number of cases of dislocation (OR = 0.52, 95% CI 0.09 to 3.08, *p* = 0.48, I^2^ = 0%, Fig. 9). Three studies [18, 28, 31] reported the occurrence of postoperative fractures. There was no significant difference between the two groups in terms of the number of fractures (OR = 1.45, 95% CI 0.27 to 7.66, *p* = 0.67, I^2^ = 0%, Fig. 10). Three studies [18–20] reported the occurrence of postoperative DVT. There was no significant difference between the two groups in terms of the number of cases of DVT (OR = 0.43, 95% CI 0.08 to 2.45, *p* = 0.34, I^2^ = 0%, Fig. 11). Two studies [18, 20] reported the occurrence of postoperative LCNT neuropraxia. There was no significant difference between the two groups in terms of the number of cases of LCNT neuropraxia (OR = 43.20, 95% CI 0.70 to 2654.71, *p* = 0.07, I^2^ = 74%, Fig. 12). Four studies [18–20, 28] reported overall number of postoperative complications. There was no significant difference between the two groups in terms of the number of overall postoperative complications (OR = 1.39, 95% CI 0.72 to 2.66, *p* = 0.32, I^2^ = 0%, Fig. 13).

### Functional outcomes

#### VAS score

**Fig. 14:**
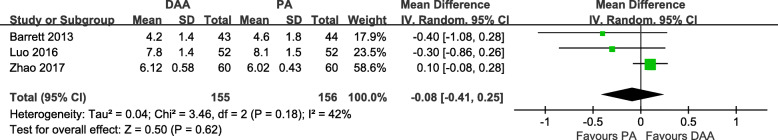
Preoperative VAS score forest plot analysis comparing the DAA vs PA in primary THA

**Fig. 15:**
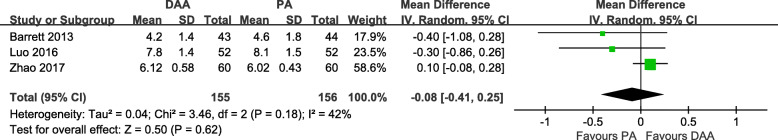
Forest plot analysis of the VAS score at 1st day postoperatively comparing the DAA vs PA in primary THA

**Fig. 16:**
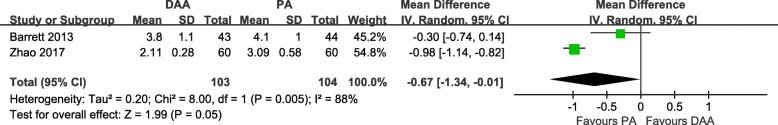
Forest plot analysis of the VAS score at 2nd days postoperatively comparing the DAA vs PA in primary THA

**Fig. 17:**
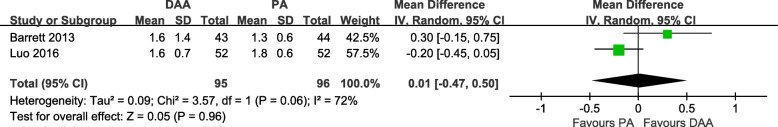
Forest plot analysis of the VAS score at 12 months postoperatively comparing the DAA vs PA in primary THA

Three studies [20, 28, 31] with a total of 311 patients were included in the comparison of the VAS score between the DAA and PA in primary THA. There was no significant difference between the two groups in terms of the preoperative VAS score (MD = -0.08, 95% CI − 0.41 to 0.25, *p* = 0.62, I^2^ = 42%, Fig. 14). Two studies [28, 31] reported the VAS score on 1st and 2nd day postoperatively. The DAA yield a significantly higher VAS score at 1st day postoperatively (MD = -0.65, − 0.91 to − 0.38, *p* < 0.00001, I^2^ = 0%, Fig. 15). The DAA showed a significantly higher VAS score at 2nd days postoperatively (MD = -0.67, − 1.34 to − 0.01, *p* = 0.05, I^2^ = 88%, Fig. 16), but there was statistically significant heterogeneity among the studies (I^2^ = 88%). Two studies [20, 28] reported the VAS score at 12 months postoperatively. There was no significant difference between the two groups in terms of the VAS score at 12 months postoperatively (MD = -0.01, 95% CI − 0.47 to 0.50, *p* = 0.96, I^2^ = 72%, Fig. 17).

#### Harris hip score (HHS)

**Fig. 18:**
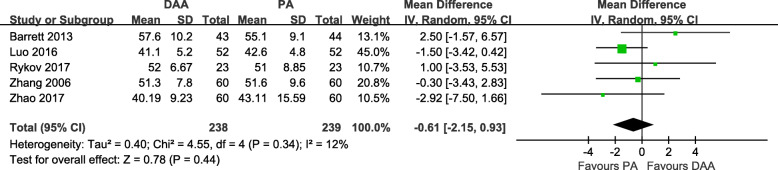
Preoperative HHS score forest plot analysis comparing the DAA vs PA in primary THA

**Fig. 19:**
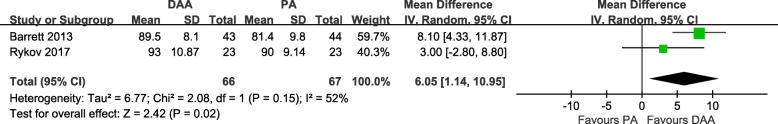
Forest plot analysis of the HHS at 6 weeks postoperatively comparing the DAA vs PA in primary THA

**Fig. 20:**
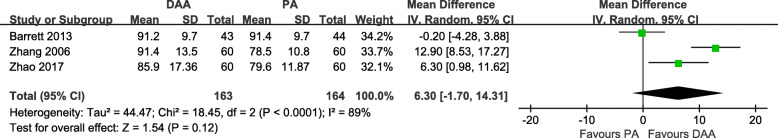
Forest plot analysis of the HHS at 3 months postoperatively comparing the DAA vs PA in primary THA

**Fig. 21:**
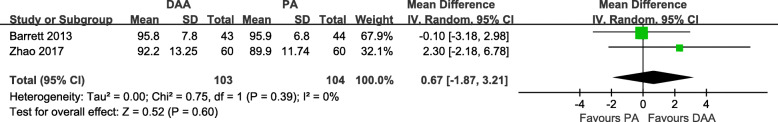
Forest plot analysis of the HHS at 6 months postoperatively comparing the DAA vs PA in primary THA

**Fig. 22:**
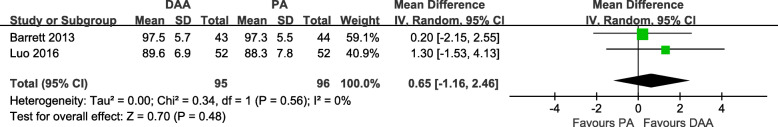
Forest plot analysis of the HHS at 12 months postoperatively comparing the DAA vs PA in primary THA

Five studies [19, 20, 28, 30, 31] with a total of 477 patients were included in the comparison of the HHS score between the DAA and PA in primary THA. There was no significant difference between the two groups in terms of the preoperative HHS score (MD = -0.61, 95% CI − 2.15 to 0.93, *p* = 0.44, I^2^ = 12%, Fig. 18). Two studies [28, 30] reported the HHS score at 6 weeks postoperatively. The DAA yield a significantly higher HHS score at 6 weeks postoperatively (MD = 6.05, 1.14 to 10.95, *p* = 0.02, I^2^ = 52%, Fig. 19). Three studies [19, 28, 31] reported the HHS score at 3 months postoperatively. There was no significant difference between the two groups in terms of the HHS score at 3 months postoperatively (MD = 6.30, 95% CI − 1.70 to 14.31, *p* = 0.12, I^2^ = 89%, Fig. 20). Two studies [28, 31] reported the HHS score at 6 months postoperatively. There was no significant difference between the two groups in terms of the HHS score at 6 months postoperatively (MD = 0.67, 95% CI − 1.87 to 3.21, *p* = 0.60, I^2^ = 0%, Fig. 21). Two studies [20, 28] reported the HHS score at 12 months postoperatively. There was no significant difference between the two groups in terms of the HHS score at 12 months postoperatively (MD = 0.65, 95% CI − 1.16 to 2.46, *p* = 0.48, I^2^ = 0%, Fig. 22).

### Radiographic outcomes

**Fig. 23:**
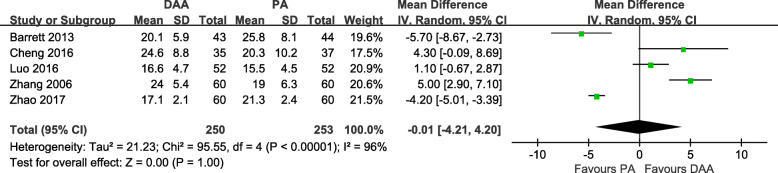
Postoperative anteversion angle forest plot analysis comparing the DAA vs PA in primary THA

**Fig. 24:**
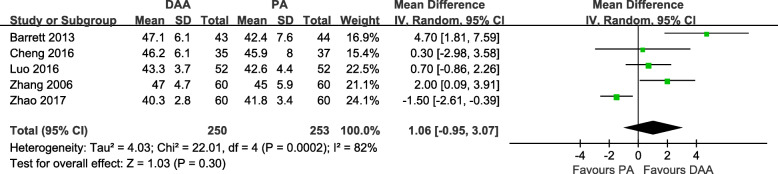
Postoperative abduction angle forest plot analysis comparing the DAA vs PA in primary THA

According to the Lewinnek safe zone (anteversion angle of 15° ± 10° and abduction angle of 40° ± 10°) [32], we estimated the radiographic outcomes of the DAA and PA. Five studies [18–20, 28, 31] with a total of 503 patients were included in the comparison of the radiographic outcomes between the DAA and PA in primary THA. There was no significant difference between the two groups in the postoperative anteversion angle (MD = -0.01, 95% CI − 4.21 to 4.20, *p* = 1.00, I^2^ = 96%, Fig. 23). Besides, there was no significant difference between the two groups in the postoperative abduction angle (MD = 1.06, 95% CI − 0.95 to 3.07, *p* = 0.30, I^2^ = 82%, Fig. 24).

## Discussion

We performed this systematic review and meta-analysis of 7 randomized controlled trials with 600 participants to compare the DAA and PA in primary THA. In the comparison of the clinical outcomes, we found that the DAA was associated with a longer surgery by a mean duration of 13.74 min (95% CI 6.88 to 20.61, *p* < 0.0001, I^2^ = 93%). There was no significant difference between the DAA and PA groups in the length of the incision, length of hospital stay (LOS), blood loss, transfusion rates or complication rates. In the comparison of functional outcomes, the early functional outcomes were significantly better in the DAA group than in the PA group, such as the visual analogue scale (VAS) score at 1st day postoperatively (MD = -0.65, 95% CI − 0.91 to − 0.38, *p* < 0.00001, I^2^ = 0%), VAS score at 2nd days postoperatively (MD = -0.67, 95% CI − 1.34 to − 0.01, *p* = 0.05, I^2^ = 88%) and Harris Hip Score (HHS) at 6 weeks postoperatively (MD = 6.05, 95% CI 1.14 to 10.95, *p* = 0.02, I^2^ = 52%). There was no significant difference between the two groups regarding the late functional outcomes, such as the VAS score at 12 months postoperatively or HHS scores at 3, 6, or 12 months postoperatively. Significant differences in the radiographic outcomes were not detected. To the best of the authors’ knowledge, this was the first meta-analysis of RCTs with direct evidence that comprehensively compared the clinical, functional and radiographic outcomes of primary total hip arthroplasty between the DAA and PA.

In contrast to meta-analyses published in the past, our study only included RCTs and compared the clinical, functional and radiographic outcomes systematically, providing level I evidence of evidence-based medicine [33]. The meta-analysis by Miller et al [24] showed a shorter incision length, less pain experienced in the hospital, a lesser need for opioid medications and a shorter LOS in the DAA group than in the PA group. However, one study [34] in their meta-analysis compared the DAA and mini-posterior approach instead of the conventional PA, which may have increased the heterogeneity. Wang et al [25] reported a significantly shorter incision length and significantly less postoperative blood loss in the DAA group than in the PA group. They also found no significant difference in the operation time or rate of complications between the two groups. However, they included one nonrandomized study and one retrospective study in the meta-analyses, which decreased the reliability of the results. Jia et al [21] found a significantly shorter LOS and significantly longer surgery duration in the DAA group than in the PA group. The authors also included a mini-posterior approach study, which may have increased the level of heterogeneity. In another meta-analysis by Miller et al [23], the DAA was found to be associated with a lower rate of infection, dislocation, and reoperation. However, most of their studies were retrospective, which inevitably led to bias. The LCNT neuropraxia outcomes varied among the studies, and only two RCTs [18, 20] included in our study reported this specific complication. Some other researchers [14, 21, 34] reported different LCNT neuropraxia outcomes in non-RCTs. We believe this high level of heterogeneity may be due to the different levels of experience among the surgeons. In our study, early functional outcomes, such as the VAS score at 1st day postoperatively, VAS score at 2nd days postoperatively and HHS at 6 weeks postoperatively, were significantly better in the DAA group than in the PA group. Some other studies [21, 22, 25] also showed better early functional outcomes and lower pain scores in the DAA group. Our findings support this conclusion and increase the level of evidence. Due to a lack of more effective data, we failed to explore functional outcomes such as the EQ. 5D, 6MWT, WOMAC and HOOS results. In a comparison of the radiographic outcomes, Jia et al [21] also found that there were little differences in the prosthetic position between the two groups.

There were nearly no statistically significant differences in the demographic characteristics of the patients in our meta-analysis. In addition, none of the studies were learning cases, which prevented this factor from influencing the results [35]. However, there was still high heterogeneity among most outcomes. We considered this result to be mainly due to the differences among the surgeons and the hospitals in how the surgical approaches were performed. The lack of a sufficient number of RCTs may be another important reason for the high heterogeneity.

This study has several limitations. First, multiple comparisons were performed in our study, which may increase the risk of type 1 error. However, most of our test results were not significant, thereby demonstrating a low risk of type 1 error inflation. Second, the number of RCTs included in the study was insufficient, which might lead to inaccurate results. Third, some RCTs used unclear or high-risk allocation concealment and selective reporting methods, which may decrease the quality of the study. Fourth, available information about complications is insufficient. Therefore, the complication outcomes were not sufficiently reliable. Finally, we could not explore the intermediate-stage functional outcomes because of a lack of sufficient data.

## Conclusion

The DAA requires a longer surgery duration than does the PA in primary total hip arthroplasty. The DAA yields improved early functional recovery compared with the PA. There was no significant difference between the two groups in terms of other clinical, complication-related, late functional or radiographic outcomes. The evidence on the superiority of the DAA is insufficient and needs to be investigated further.

## Data Availability

All data and materials are contained within the manuscript.

## Abbreviations

RCTs: Randomized controlled trials
DAA: Direct anterior approach
PA: Posterior approach
THA: Total hip arthroplasty
VAS: Visual analogue scale
MD: Mean deviation
CI: Confidence interval
HHS: Harris hip score
LOS: Length of stay
OA: Osteoarthritis
PRISMA: Preferred reporting items for systematic reviews and meta-analyses
LCNT: Lateral cutaneous nerve of the thigh
USA: The United States of America
BMI: Body mass index
OR: Odds ratio
6MWT: The 6-min walk test
WOMAC: The Western Ontario and McMaster Universities Osteoarthritis Index
HOOS: Hip disability, and osteoarthritis outcome score
DVT: Deep vein thrombosis
